# A biomechanical comparison of all-inside cruciate ligament graft preparation techniques

**DOI:** 10.1186/s40634-018-0158-0

**Published:** 2018-10-10

**Authors:** Colter R Wichern, Kathryn C Skoglund, Joseph G O’Sullivan, Anora K Burwell, Joseph T Nguyen, Andrea Herzka, Jacqueline M Brady

**Affiliations:** Department of Orthopaedics and Rehabilitation, Oregon Health and Science University, Portland, OR USA

**Keywords:** ACL, PCL, Ligament reconstruction, Graft preparation

## Abstract

**Background:**

The all-inside cruciate ligament graft preparation technique has become popular due to its utility in sparing a growing physis, preserving a tendon in ACL surgery, and/or reduction of pain. However, few studies have compared graft preparation techniques to determine the ideal construct for cruciate ligament reconstruction. We sought to compare biomechanical properties of two quadrupled all-inside cruciate ligament graft preparation techniques and three alternative all-inside graft preparation techniques that may be used when the available tendon is too short to be quadrupled.

**Methods:**

Fifty porcine extensor tendons were evenly divided into five groups (*n* = 10) representing all-inside graft preparation techniques, including two quadrupled (Quad-A, Quad-B) and three alternative methods (Tripled, Folded, Two-Doubled). Each graft construct underwent preconditioning (10 loading cycles from 20 to 50 N at 0.1 Hz), cyclic loading (500 loading cycles from 50 to 250 N at 1.0 Hz) and load-to-failure (tension applied at 20 mm/min).

**Results:**

Quad-A and Quad-B demonstrated no significant differences in cyclic displacement (10.5 ± 0.3 vs 11.7 ± 0.4 mm; *p* = 0.915), cyclic stiffness (1086.2 ± 487.3 vs 460.4 ± 71.4 N/mm; *p* = 0.290), pullout stiffness (15.9 ± 4.3 vs 7.4 ± 4.4 N/mm; *p* = 0.443), ultimate failure load (641.2 ± 84.7 vs 405.9 ± 237.4 N; *p* = 0.672), or ultimate failure displacement (47.3 ± 6.7 vs 55.5 ± 0.7 mm; *p* = 0.778). The mean cyclic displacement of the Two-Doubled group was significantly greater than the Quad-A (29.7 ± 2.2 vs 10.5 ± 0.3 mm; *p* < 0.001), Quad-B (29.7 ± 2.2 vs 11.7 ± 0.4 mm; *p* < 0.001), Tripled (29.7 ± 2.2 vs 11.3 ± 0.2 mm; *p* < 0.001), and Folded group (29.7 ± 2.2 vs 13.3 ± 0.2 mm; *p* < 0.001). There were no other statistically significant differences between the three alternative all-inside graft preparation techniques.

**Conclusion:**

The current study demonstrates the biomechanical properties of two quadrupled all-inside graft constructs, Quad-A and Quad-B, are not significantly different. When the available tendon is of insufficient length, the Two-Doubled group demonstrated more than twice the cyclic displacement of all other graft preparation techniques, and is therefore not recommended for use in all-inside cruciate ligament reconstruction.

## Background

The all-inside technique is a relatively new approach to anterior cruciate ligament (ACL) and posterior cruciate ligament (PCL) reconstruction that has gained popularity in recent years, in part because of its minimally invasive approach, preservation of cortical bone, and potential sparing of tendon tissue (autograft) or cost (allograft) compared to traditional techniques (Benea et al., 2014; Smith et al., 2008; Connaughton et al., 2017; Jones and Schuett, 2018). This technique can also be employed to preserve the integrity of the tibial and femoral physes by use of sockets that are restricted to the epiphysis, a feature that is particularly important for ACL reconstruction in skeletally immature patients (Stadelmaier et al., 1995; Kocher et al., 2002; Kercher et al., 2009; Frosch et al., 2010; Cordasco et al., 2016). Fashioned with either an autograft or allograft tendon, which is prepared and secured within femoral and tibial sockets using cortical suspension devices, the all-inside ACL and PCL reconstruction technique incorporates a construct with multiple components susceptible to failure. Accordingly, careful research on the relative efficacy and biomechanical properties of these techniques is needed.

While various graft fixation constructs have been studied in depth (Fabbri et al., 2016; Lubowitz, 2012; Fritsch et al., 2017; Sasho et al., 2018; Tiefenboeck et al., 2018), current biomechanical literature falls short in offering relative comparisons between graft preparation techniques. Although the quadrupled graft preparation technique is the preferred method of graft preparation, multiple techniques have been described (Lubowitz, 2012; Fritsch et al., 2017; Sasho et al., 2018; Tiefenboeck et al., 2018; McCarthy et al., 2012). Additionally, insufficient tendon length can become a problem for ACL reconstruction in petite patients or in the case of inadvertent amputation of a tendon during harvest, such as in the setting of previous surgery and resultant scar. In the setting of PCL reconstruction, tendon length is a relatively consistent concern because a longer graft is required to approximate the deficient ligamentous structure. In these cases, the harvested tendon or allograft tendon may be of insufficient length to create a quadrupled graft of appropriate length, and one of several alternative methods for graft preparation may be used. In one commonly used alternative, the graft is tripled, and a whipstitch placed at each end as a result. Another involves selection of two shorter grafts, and doubling them. Finally, a different tendon graft might be used and folded back onto itself (such as Achilles tendon).

**Fig. 1:**
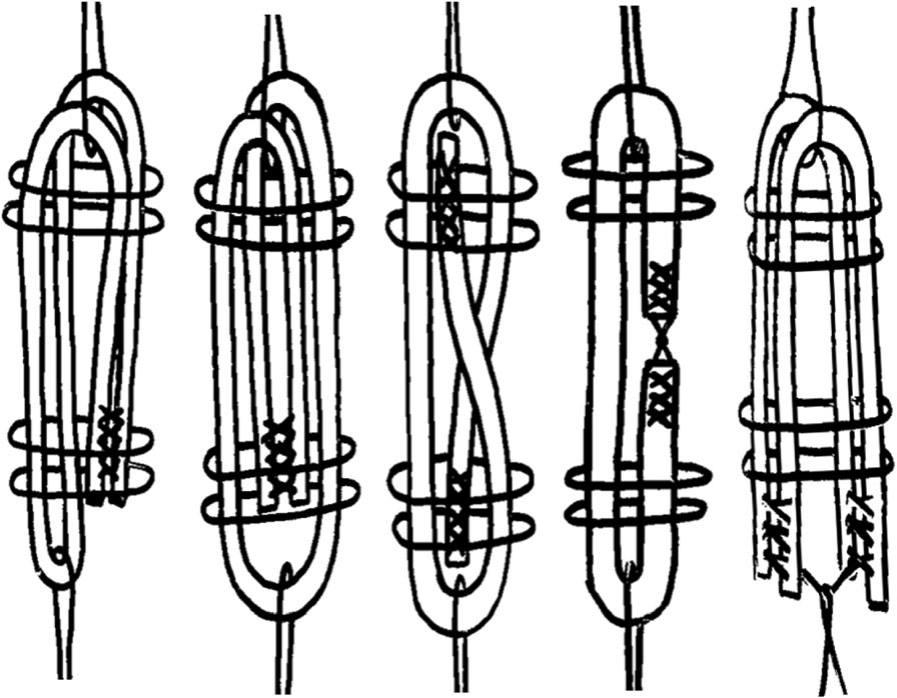
Graft preparation techniques illustrated from left-to-right include: Quad-A: the two ends of the single graft are doubled over one suspensory suture, whipstitched together, and then passed together around another suspensory suture together. Quad-B: the two ends are passed in opposite directions around the second suspensory suture before the whipstitch is placed. Tripled: the graft ends are whipstitched separately, then each is passed around a suspensory suture, resulting in a whipstitch at each end of the construct. Folded: a single graft is whipstitched at each end, and the ends are each passed around a suspensory suture, meeting in the middle of the construct. Two-Doubled: two grafts are selected, and doubled over a suspensory suture. The whipstitched ends are then secured to a separate suspensory suture by way of knots tied in the whipstitch tails. The femoral end of each graft is depicted at the top of the schematic, and the tibial end is at the bottom

The purpose of the current study was to examine the biomechanical properties of five different methods used to prepare grafts for all-inside ACL and PCL reconstruction: two types of quadrupled constructs (Quad-A and Quad-B), a tripled construct (Tripled), a folded construct (Folded), and two grafts in doubled form (Two-Doubled) which are illustrated in Fig. 1. The two quadrupled constructs represent the preferred techniques used for graft preparation in all-inside cruciate ligament reconstruction. The remaining three constructs are potential alternative methods used for construction of grafts when the available tendon is not long enough to produce a quadrupled graft of adequate length. We hypothesized that, while the two quadrupled techniques would demonstrate similar biomechanical properties, the three alternative techniques would demonstrate significant differences in their biomechanical characteristics.

## Methods

### Porcine extensor tendons

Fifty fresh frozen (−23^o^ C) porcine extensor tendons, acquired from J & J Packing Co. Inc. (Brookshire, TX) were randomly divided into five groups. Specimens were thawed in a refrigerator within 48 h of use and underwent only one freeze-thaw cycle. Once thawed, the bifurcate tendon was divided, all adherent muscle tissue was removed, and grafts were trimmed to achieve average graft construct lengths of 50–70 mm for all groups (Table 1). In an effort to maintain structural integrity of the extensor tendons, individual tendons were not trimmed longitudinally to create smaller tendon widths. Specimens were kept hydrated by being wrapped in a moist paper towel during preparation and were sprayed with water approximately every two minutes during testing.

**Table 1:** Dimensions of porcine extensor tendons and ACL/PCL Graft constructs; *n* = 10 in each graft group

Graft type	Tendon length (mm)	Tendon width (mm)	Graft construct length (mm)	Graft construct width (mm)
Quad-A	195.9 ± 6.7	12.5 ± 0.4	57.0 ± 2.2	16.5 ± 0.3
Quad-B	200.2 ± 5.1	11.5 ± 0.3	52.5 ± 1.9	17.2 ± 0.7
Tripled	196.3 ± 2.5	11.3 ± 0.3	65.9 ± 1.2	15.3 ± 0.3
Folded	148.5 ± 1.1	10.8 ± 0.3	68.9 ± 1.0	12.6 ± 0.4
Two-Doubled	115.5 ± 1.2	11.2 ± 0.3	60.3 ± 1.0	17.8 ± 0.7

All data reported as mean ± SEM

### Surgical techniques

The tendons were prepared using one of the five techniques illustrated in Fig. 1. All graft constructs were prepared either by, or under the direct supervision of, the same faculty orthopaedic surgeon. One of the coauthors served as an assistant during graft preparation, to ensure consistency of technique. The graft constructs were suspended with double-looped No. 5 Fiberwire suture on both the femoral and tibial ends. The final dimensions of the graft constructs in each of the groups are summarized in Table 1.

#### Quadrupled techniques

The first quadrupled technique, Quad-A, was prepared by wrapping the tendon around the No. 5 suture in the same fashion that has been demonstrated by McCarthy and colleagues (McCarthy et al., 2012). The tendon was folded in half around one No. 5 Fiberwire suture loop at the tibial end, the free ends were then whip-stitched together using a No.2 Fiberwire suture and passed together in the same direction around the second No. 5 suture loop at the femoral end. The free ends were tucked into the fold on the tibial end, with the whip stitch suture wrapped and tied around the tibial suspensory suture. The graft was secured with four cerclage stitches, two on the femoral end and two on the tibial end, passing through each of the four tendon limbs and secured with buried knots.

The second quadrupled technique, Quad-B, was prepared by wrapping the tendon around the No. 5 suture in the same fashion that has been demonstrated by Lubowitz (Lubowitz, 2012). The tendon was folded in half around a No. 5 suture loop at the tibial end and the free ends were passed in opposite directions around the No. 5 suture loop on the femoral end. The free tendon ends were then whip-stitched together with a No. 2 suture and tucked into the folded tendon at the tibial end, with the whip stitch suture wrapped and tied around the tibial suspensory suture. The graft was secured with four cerclage stitches, two on the femoral end and two on the tibial end, passing through each of the four tendon limbs and secured with buried knots.

#### Tripled technique

The Tripled graft construct was created by whip-stitching each of the free ends with a No. 2 suture, passing each free end around a No. 5 suture and then tucking the ends into the opposite femoral and tibial folds. The No. 2 suture from the whip-stitched ends was then wrapped and knotted around the suspensory suture at the tibial and femoral ends, respectively. The graft was secured with four cerclage stitches, two on the femoral end and two on the tibial end, passing through each of the three tendon limbs, and secured with buried knots.

#### Folded technique

The Folded graft construct was created by passing one of the free ends of the tendon around the femoral or tibial-sided No. 5 suture, and wrapping around so the free ends met in the center of the graft. Each free end was then whip-stitched to the tendon on the opposing long side of the graft using a No. 2 suture. The graft was secured with four cerclage stitches, two on the femoral end and two on the tibial end, passing through both of the tendon limbs and secured with buried knots.

#### Two-doubled technique

The Two-Doubled technique was prepared using two tendons, each folded over a No. 5 looped-suture to produce a total of four tendon limbs. The free-ends were whip-stitched together and the suture connecting the two tendons was passed through a second No. 5 looped-suture on the tibial end. The graft was secured with four cerclage stitches, two on the femoral end and two on the tibial end, passing through each of the four tendon limbs, and secured with buried knots.

### Biomechanical testing

**Fig. 2:**
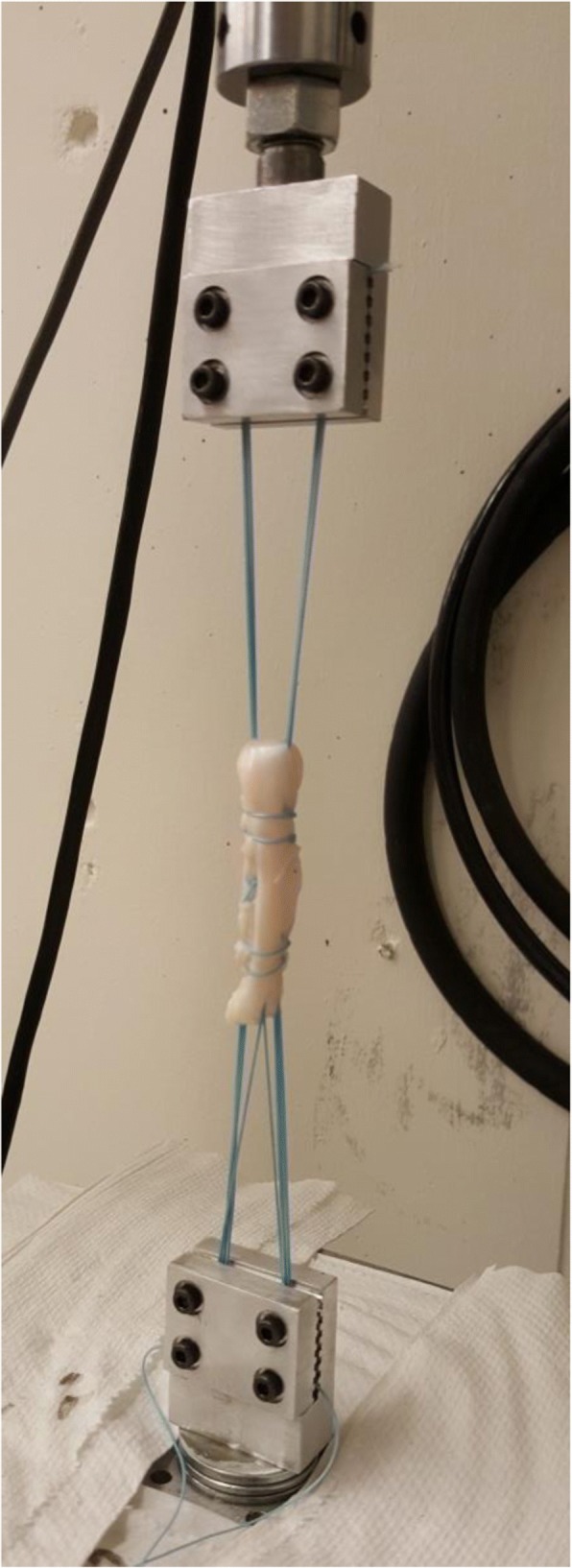
A quadrupled tendon suspended using No. 5 suture from clamps in the MTS machine

Graft constructs were mounted onto an 858 Mini Bionix Material Testing System (MTS Systems Corp., Eden Prairie, MN) by passing the doubled No. 5 looped-suture in a figure-of-eight pattern around 4 screws which were used to lightly compress aluminum clamps on the suture on both the femoral and tibial ends (Fig. 2).

Load and displacement data were collected at a frequency of 20 Hz during all phases of testing and the testing protocol was executed as previously described by Walsh and colleagues (Walsh et al., 2008). Displacement was measured by the elongation of the distance between clamps during testing. Prior to testing each specimen, the load cell was zeroed without tension. Preloading occurred as tension was applied to each specimen at a rate of 1 N/sec up to 20 N and displacement was zeroed while the load was held at 20 N for 10–20 s. Preconditioning was performed with 10 loading cycles from 20 to 50 N at 0.1 Hz and subsequent cyclic loading occurred for 500 loading cycles from 50 to 250 N at 1.0 Hz. Cyclic displacement was defined as the displacement between clamps occurring during cyclic testing. Cyclic stiffness was defined as the slope of the load versus displacement curve in the elastic region during the cyclic testing.

To test load-to-failure, tension was applied to each specimen at a rate of 20 mm/min until the tendon or the suture failed, producing a rapid decrease in load. Pullout stiffness was defined as the slope of the load versus displacement curve in the elastic region during the load-to-failure testing. Ultimate failure load was defined as the load at the onset of plastic deformation within the system, based on the change in slope of the load versus displacement curve, regardless of the source of that failure. Ultimate failure displacement was defined as the amount of displacement within the system at the point of ultimate failure load.

### Statistical analysis

All outcomes were assessed using generalized estimating equation (GEE) models to compare the differences of the graft types while controlling for the clustered nature of the data. GEE parameter estimates were generated using quasi-likelihood estimation. Post hoc tests were conducted to determine differences between graft types. Reported *p*-values were Bonferroni-adjusted to account for the type I error from multiple comparisons. Statistical significance was defined as a *p*-value < 0.05. All statistical analyses were performed using SAS version 9.2 (SAS Institute; Cary, NC).

## Results

### Tendon and graft construct dimensions

The dimensions of the tendons and graft constructs for each of the preparation techniques, prior to testing, are reported in Table 1 with the associated post hoc pairwise comparisons reported in Table 2. No significant differences were found in the dimensions of the Quad-A vs Quad-B groups in terms of tendon length (*p* > 0.999), tendon width (*p* = 0.373), graft construct length (*p* = 0.439), or graft construct width (*p* > 0.999).

**Table 2:** Post hoc pairwise comparisons of tendon & graft construct dimensions

Parameter	Graft type	Quad-A	Quad-B	Tripled	Folded	Two-Doubled
Tendon length	Quad-A		> 0.999	> 0.999	**< 0.001**	**< 0.001**
	Quad-B	> 0.999		> 0.999	**< 0.001**	**< 0.001**
	Tripled	> 0.999	> 0.999		**< 0.001**	**< 0.001**
	Folded	**< 0.001**	**< 0.001**	**< 0.001**		**< 0.001**
	Two-Doubled	**< 0.001**	**< 0.001**	**< 0.001**	**< 0.001**	
Tendon width	Quad-A		0.373	0.132	**0.006**	**0.018**
	Quad-B	0.373		> 0.999	> 0.999	> 0.999
	Tripled	0.132	> 0.999		> 0.999	> 0.999
	Folded	**0.006**	> 0.999	> 0.999		> 0.999
	Two-Doubled	**0.018**	> 0.999	> 0.999	> 0.999	
Graft construct length	Quad-A		0.439	**0.002**	**< 0.001**	> 0.999
	Quad-B	0.439		**< 0.001**	**< 0.001**	**0.008**
	Tripled	**0.002**	**< 0.001**		> 0.999	0.132
	Folded	**< 0.001**	**< 0.001**	> 0.999		**0.003**
	Two-Doubled	> 0.999	**0.008**	0.132	**0.003**	
Graft construct width	Quad-A		> 0.999	0.990	**< 0.001**	0.747
	Quad-B	> 0.999		0.106	**< 0.001**	> 0.999
	Tripled	0.990	0.106		**0.004**	**0.010**
	Folded	**< 0.001**	**< 0.001**	**0.004**		**< 0.001**
	Two-Doubled	0.747	> 0.999	**0.010**	**< 0.001**	

All data reported as *p*-values. Statistical significance was set at the 0.05 level

After being trimmed to achieve final graft construct lengths of 50–70 mm, the tendon lengths of the Tripled (196.3 mm), Folded (148.5 mm) and Two-Doubled group (115.5 mm) demonstrated significant differences when compared to one another (*p* < 0.001). There were no statistically significant differences (*p* > 0.05) between the individual tendon widths of the Tripled, Folded and Two-Doubled groups. The graft construct length of the Tripled vs Folded groups was not significantly different (*p* > 0.999), nor was the difference between the Tripled vs Two-Doubled groups (*p* = 0.132). There was a statistically significant difference between the Folded vs Two-Doubled group (68.9 ± 1.0 vs 60.3 ± 1.0 mm; *p* = 0.003), however. The final graft construct widths, prior to testing, demonstrated statistically significant differences in the Tripled vs Folded (1*p* = 0.004), Tripled vs Two-Doubled (1*p* = 0.010) and Folded vs Two-Doubled (*p* < 0.001) group comparisons.

### Biomechanical testing

The biomechanical parameter estimates of each of the graft preparation techniques are reported in Table 3. Quad-A vs Quad-B comparisons demonstrated no significant differences in cyclic displacement (*p* = 0.915), cyclic stiffness (*p* = 0.290), pullout stiffness (*p* = 0.443), ultimate failure load (*p* = 0.672), or ultimate failure displacement (*p* = 0.778). When compared to the Tripled group, the Quad-A group demonstrated significantly greater ultimate failure load (*p* = 0.046) and less ultimate failure displacement (*p* = 0.010). The post hoc pairwise comparisons for each of the biomechanical parameters are organized in Table 4.

**Table 3:** Biomechanical Properties of ACL/PCL Graft Constructs; *n* = 10 in each graft group

Graft type	Cyclic displacement (mm)	Cyclic stiffness (N/mm)	Pullout stiffness (N/mm)	Ultimate failure load (N)	Ultimate failure displacement (mm)
Quad-A	10.5 ± 0.3	1086.2 ± 487.3	15.9 ± 4.3	641.2 ± 84.7	47.3 ± 6.7
Quad-B	11.7 ± 0.4	460.4 ± 71.4	7.4 ± 4.4	405.9 ± 237.4	55.5 ± 0.7
Tripled	11.3 ± 0.2	385.4 ± 48.0	0.9 ± 0.8	73.3 ± 59.7	76.4 ± 0.6
Folded	13.3 ± 0.2	243.5 ± 36.1	2.1 ± 2.0	143.4 ± 140.0	69.7 ± 0.5
Two-Doubled	29.7 ± 2.2	210.5 ± 47.2	2.0 ± 1.6	128.6 ± 108.3	55.4 ± 6.1

All data reported as mean ± SEM

**Table 4:** Post hoc pairwise comparisons of biomechanical properties

Biomechanical property	Graft type	Quad-A	Quad-B	Tripled	Folded	Two-Doubled
Cyclic displacement	Quad-A		0.915	0.979	0.334	**< 0.001**
	Quad-B	0.915		0.999	0.830	**< 0.001**
	Tripled	0.979	0.999		0.677	**< 0.001**
	Folded	0.334	0.830	0.677		**< 0.001**
	Two-Doubled	**< 0.001**	**< 0.001**	**< 0.001**	**< 0.001**	
Cyclic stiffness	Quad-A		0.290	0.190	0.074	0.058
	Quad-B	0.290		0.999	0.958	0.931
	Tripled	0.190	0.999		0.991	0.981
	Folded	0.074	0.958	0.991		> 0.999
	Two-Doubled	0.058	0.931	0.981	> 0.999	
Pullout stiffness	Quad-A		0.443	0.059	0.087	0.084
	Quad-B	0.443		0.752	0.853	0.847
	Tripled	0.059	0.752		> 0.999	> 0.999
	Folded	0.087	0.853	> 0.999		> 0.999
	Two-Doubled	0.084	0.847	> 0.999	> 0.999	
Ultimate failure load	Quad-A		0.672	**0.046**	0.089	0.078
	Quad-B	0.672		0.470	0.673	0.630
	Tripled	**0.046**	0.470		0.996	0.998
	Folded	0.089	0.673	0.996		> 0.999
	Two-Doubled	0.084	0.630	0.998	> 0.999	
Ultimate failure displacement	Quad-A		0.778	**0.010**	0.053	0.788
	Quad-B	0.778		0.123	0.419	> 0.999
	Tripled	**0.010**	0.123		0.910	0.120
	Folded	0.053	0.419	0.910		0.410
	Two-Doubled	0.788	> 0.999	0.120	0.140	

All data reported as *p*-values. Statistical significance was set at the 0.05 level

The mean cyclic displacement of the Two-Doubled group was the largest amongst all groups, and was significantly greater than the Quad-A group (*p* < 0.001), Quad-B group (*p* < 0.001), Tripled group (*p* < 0.001), and Folded group (*p* < 0.001). No other statistically significant differences were observed between the groups. However, the pullout stiffness of the Quad-A group was nearly statistically significantly greater than the Tripled group (*p* = 0.059).The mechanisms of failure of the various graft types were also observed. No slipping of the suspensory suture at the clamp-suture junction was observed, nor was stretching of the Fiberwire suture. Quad-A and Quad-B grafts failed when the whipstitched free end of the graft pulled from its tibial end and through the nearest cerclage suture, elongating the graft significantly. The Tripled group showed a similar mechanism of failure as the free, whipstitched ends at the femoral and tibial sides of the graft pulled through the cerclage sutures.

The mechanism of failure in the Folded group was more variable: four grafts failed when the whipstitch suture itself broke, three grafts failed when the whipstitched end pulled through the cerclage suture, and three grafts failed when the cerclage sutures broke at the tibial end of the graft.

The mechanism of failure of the Two-Doubled group was consistently located at the tibial side of the graft where no tendon tissue was wrapped around suspensory suture, instead, the whip stitch was looped around the suspensory suture. In seven cases, the whipstitch broke at the tibial end of the graft. Two grafts failed when the whipstitch slid through the tendon, and one graft failed when the whipstitch broke at its interface with the suspensory suture.

## Discussion

We determined that the biomechanical properties of two quadrupled cruciate ligament graft constructs, Quad-A and Quad-B, are not significantly different. To our knowledge, this is the first biomechanical comparison of these two commonly used techniques. We also demonstrated that these two quadrupled techniques achieve ultimate failure loads that are sufficient to withstand the forces (150–590 N) encountered during early rehabilitation protocols following ACL and PCL reconstruction (Shelburne et al., 2004). Thus, our results support either of the quadrupled techniques being the preferred method of graft preparation for all-inside ACL and PCL reconstruction.

Importantly, our results indicate that the least robust portion of an all-inside graft construct is the suture used to secure it and/or suspend it. This is in contrast to other graft preparation techniques, in which the graft itself is the first to fail when tested in a biomechanical environment. The all-inside graft preparation technique seems to transfer the loads from the fixation itself to the whipstitch.

At present, the literature remains unclear on which graft preparation technique should be used if the tendon is of insufficient length to produce a quadrupled graft. In the current study, we compared three alternative methods of graft preparation to the quadrupled techniques to evaluate their relative biomechanical strength. During cyclic loading, cyclic displacement of the Two-Doubled group was greater than twice that of every other group, which makes this technique the least appealing option for all-inside ACL and PCL reconstruction. Although not statistically significant, due likely to the high variability of the Qual-A and Quad-B groups relative to the other groups, the Tripled technique demonstrated the least amount of cyclic displacement of the three alternative methods. Thus the Tripled group may be the preferred technique to employ if the tendon is of insufficient length to be quadrupled. However, further research is needed to clarify if this is truly the case. The Tripled group failed at significantly lower ultimate failure loads when compared to the Quad-A group, but not the Quad-B group; a significance that was lost in the Folded and Two-Doubled groups as a result of higher variability. Ultimate failure displacement was also significantly greater in the Tripled group compared to the Quad-A group; however, this parameter has less clinical significance due to the anatomical limitations of the knee joint itself.

While the ultimate failure displacement in the Two-Doubled group did not differ significantly, the variability of this group was relatively high when compared to the Tripled and Folded groups. This appeared to be due to failure of the suture material at the tibial end of the graft where the free ends were whipstitched together, and this whipstitch was utilized to incorporate the suspensory suture. This may indicate that wrapping the tendon itself, rather than suture, around the suspensory device lends inherent strength to the graft construct. Therefore, we question the integrity of graft preparation in single tendon, non-folded constructs such as quadriceps tendon mounted on a suspensory device via suture alone on the both the femoral and tibial ends (Slone et al., 2016).

Ensuring that various graft fixation methods are sufficient to withstand the forces experienced during early post-operative rehabilitation has been an active area of research in recent years. Fortunately, biomechanical studies have demonstrated that many suspensory devices have sufficient strength to withstand forces that exceed even the highest loads experienced during early exercise protocols (Bartz et al., 2007; Johnson et al., 2015; Rylander et al., 2014). Additionally, there are several different approaches used for graft fixation in all-inside reconstruction, including interference screws, suture buttons and a combination of interference screws and suture buttons together (Frosch et al., 2010). As cruciate ligament reconstruction trends towards less invasive techniques such as all-inside ACL and PCL replacement, it becomes increasingly important to evaluate the biomechanical properties of the graft constructs as well as the fixation devices.

Johnson et al. demonstrated that modern cortical suspension devices are capable of withstanding ultimate forces of 784–2231 N (Johnson et al., 2015). These forces are much greater than those tolerated by the tissue grafts in our study, which suggests that the suture by which the graft is secured, or to the fixation device is the weakest component of the graft-link construct. This extrapolation is complicated by a variety of factors including dynamic in vivo force vectors and loads as well as by supplemental fixation techniques such as cortical suspension devices used in combination with an interference screw. Nevertheless, it is essential to identify and minimize those components which are susceptible to rupture or failure.

Fabbri et al. used a similar model to evaluate whether graft length is critical for success in all-inside reconstruction, including a quadrupled model similar to Quad-A in our study, as well as a tripled model similar to that in our study, and a half-quadrupled model that was unique to their study (Fabbri et al., 2016). The quadrupled method achieved the highest ultimate failure load, followed by the tripled method The mechanism of failure seen in their study was consistent with that seen in our study, with failure occurring by slippage of suture within the graft. This study, along with ours, provides more comprehensive investigation of quadrupled graft preparation techniques and of alternative techniques.

Mayr et al. compared several all-inside graft preparation techniques with varying degrees of suture fixation (Mayr et al., 2016). This included one model with four buried cerclage sutures (two sutures at both ends of the graft as described originally by (Lubowitz, 2012)), one with two buried cerclage sutures at the tibial end, and the final with two cerclage sutures at the tibial end with additional suspension on the tibial cortical button. The four-suture and hybrid two-suture with additional suspension model had comparable graft elongation and ultimate failure loads, thus proposing a faster graft preparation technique with comparable biomechanical strength to the current standard. Similarly, we used a hybrid suspensory device at the tibial end of the grafts by wrapping and tying the whipstitched suture around the suspensory suture. However, we chose not to focus on the level of suture burden of the various techniques, but instead the different methods of folding the grafts into various constructs.

Barbosa et al. described an alternative technique which minimizes suture in the bony socket, proposing that suture wrapping the tendon in the bony socket reduces the bone-tendon contact area crucial for biologic integration of the graft (Barbosa et al., 2017). Their technique eliminates the cerclage suture at both ends of the graft in favor of directly suturing the whip-stitched free ends to the Fiber Loop. Unfortunately, a biomechanical evaluation of the technique was not conducted, which would be an important consideration in the clinical use of this model.

Our study is not without its limitations. First, a study of porcine tendons cannot be directly compared with studies using human tissue, although our graft preparation technique was equivalent to the clinical setting. Porcine tendon specimens have been reported to have similar properties to human tendons in previous biomechanical studies (Yamanaka et al., 1999; Dargel et al., 2009) and the porcine knee is widely used as a model to investigate ACL reconstruction (Dargel et al., 2009; Debandi et al., 2011; Zhu et al., 2018). Second, the length of the prepared grafts was not uniform. While the displacement of the constructs could theoretically be affected by significant differences in width and/or length, we observed that the majority of displacement and subsequent failure occurred at the tendon/suture interface in each of the five groups. This is especially true for the Two-Doubled group, where the constructs elongated as a consequence of the whipstitch being pulled away from the free ends. Thus, it stands to reason that differences in displacement, stiffness, and load to failure are more the result of differences in the interaction between the tendon and suture rather than the material properties themselves.

Another limitation is that this study was designed to test the relative biomechanical properties of different graft preparation techniques in isolation, which does not provide information about the graft and fixation device interaction or complex as an entire unit. Additionally, the testing protocol provides information about the relative displacement, stiffness and load tolerance in a linear fashion, which limits us from making inferences based upon the external forces and dynamic vectors that are placed upon the ACL or PCL graft as it occurs in vivo. Further research is necessary to investigate the biomechanical properties of the entire graft constructs with various fixation devices in vitro in addition to appropriate prospective studies designed to identify clinically relevant outcomes in the long term.

## Conclusion

The two quadrupled techniques for all-inside cruciate ligament reconstruction demonstrated no significant difference in any of the primary endpoints measured, suggesting that either technique could be the preferred method for all-inside ACL and PCL reconstruction. The Tripled technique is preferred if a graft is of insufficient length for quadrupled preparation. The Two-Doubled technique was observed to have greater than twice the cyclic displacement of every other preparation technique tested, and is therefore not recommended for use in all-inside cruciate ligament reconstruction.
